# Seeking the Roles for Fungal Small-Secreted Proteins in Affecting Saprophytic Lifestyles

**DOI:** 10.3389/fmicb.2020.00455

**Published:** 2020-03-24

**Authors:** Daria Feldman, Oded Yarden, Yitzhak Hadar

**Affiliations:** Department of Plant Pathology and Microbiology, The R.H. Smith Faculty Agriculture, Food and Environment, The Hebrew University of Jerusalem, Jerusalem, Israel

**Keywords:** small secreted proteins, lifestyle, saprophytes, *Pleurotus*, effector

## Abstract

Small secreted proteins (SSPs) comprise 40–60% of the total fungal secretome and are present in fungi of all phylogenetic groups, representing the entire spectrum of lifestyles. They are characteristically shorter than 300 amino acids in length and have a signal peptide. The majority of SSPs are coded by orphan genes, which lack known domains or similarities to known protein sequences. Effectors are a group of SSPs that have been investigated extensively in fungi that interact with living hosts, either pathogens or mutualistic systems. They are involved in suppressing the host defense response and altering its physiology. Here, we aim to delineate some of the potential roles of SSPs in saprotrophic fungi, that have been bioinformatically predicted as effectors, and termed in this mini-review as “effector-like” proteins. The effector-like Ssp1 from the white-rot fungus *Pleurotus ostreatus* is presented as a case study, and its potential role in regulating the ligninolytic system, secondary metabolism, development, and fruiting body initiation are discussed. We propose that deciphering the nature of effector-like SSPs will contribute to our understanding of development and communication in saprophytic fungi, as well as help, to elucidate the origin, regulation, and mechanisms of fungal-host, fungal-fungal, and fungal-bacterial interactions.

## Introduction

The success of fungi to adapt and proliferate in diverse ecological niches is dependent on their ability to respond to changes in the environment. The rich, yet versatile, fungal secretome is an important component that facilitates the fast adjustment to changes occurring in the vicinity of the growing and developing fungus (Krijger et al., 2014; McCotter et al., 2016). The size of the secretome is 4–15% of the total gene number (Girard et al., 2013; Pellegrin et al., 2015). Nonetheless, the composition of fungal secretomes can be extremely variable, even within the same fungal species and under similar environmental conditions (Girard et al., 2013; Krijger et al., 2014). The secretome is composed of proteins that participate in organic matter degradation, such as proteases, lipases, Carbohydrate-Active enZymes (CAZymes), but also hydrophobins and small-secreted proteins (SSPs) (Alfaro et al., 2014; Pellegrin et al., 2015).

SSPs are secreted by fungi independently of their lifestyle and are defined as proteins that contain a signal peptide and a sequence of less than 300 amino acids. Many SSPs are coded by orphan genes, lacking known PFAM domains or similarities to known sequences. The proportion of SSPs ranges from 40 to 60% of the secretome across all lifestyles and phylogenetic groups within the fungal kingdom (Pellegrin et al., 2015; Kim et al., 2016). SSPs have been predominantly investigated in fungi that interact with living hosts, with emphasis on cysteine-rich sub-group referred to as “effectors,” key factors of infection which can suppress host defense responses as well as modulate its physiology (Veneault-Fourrey and Martin, 2011; Win et al., 2012; Alfaro et al., 2014).

Here, we follow the common paradigm defining effectors as SSPs used by a range of pathogenic and beneficial fungi to alter the physiological status of the host plant (Veneault-Fourrey and Martin, 2011; Win et al., 2012; Alfaro et al., 2014; Lo Presti et al., 2015). We specifically focus on SSPs in fungi that exhibit predominantly saprotrophic lifestyles and can be bioinformatically classified as effector (Sperschneider et al., 2016, 2018). For the sake of clarification, we will use the term “effector” to describe proteins clearly involved in fungal-host interactions, and “effector-like” for those that are classified as effectors according to Sperschneider et al. (2018), yet have been found in saprophytes. Others have already suggested that effector-like proteins may have yet undiscovered roles in saprophytic growth, nutrition and development (Girard et al., 2013; Kim et al., 2016).

## SSPs as Effectors in Fungal-Host Interactions

SSPs functioning as effectors are common in several plant pathogens (Lo Presti et al., 2015). Many effectors have weak or no sequence similarity to proteins with known activity, and only some of them are considered conserved. Nonetheless, more recent studies have revealed that effectors probably share more structural conservation, which is less evident on the basis of their primary sequences (Lo Presti et al., 2015; Selin et al., 2016; Franceschetti et al., 2017; Lo Presti and Kahmann, 2017; Plissonneau et al., 2017). One proposed explanation for this is that they are under strong selective pressure which has led to their accelerated divergent evolution (Carvunis et al., 2012; Lo Presti et al., 2015; Plissonneau et al., 2017). Some effector families were probably expanded by duplication and diversification from a common ancestor, or through horizontal gene transfer (Lo Presti et al., 2015). One criterion for classifying effectors is based on their localization in the host. They are considered either apoplastic (localize to the apoplast) or cytoplasmic (enter into the plant cells) (Lo Presti et al., 2015; Franceschetti et al., 2017). Their translocation into the plant cells is still poorly understood (Lo Presti and Kahmann, 2017).

The content of small secreted cysteine-rich proteins, usually classified as effectors, is relatively close between saprophytes and plant pathogens, and lower in animal pathogens (Krijger et al., 2014). Genomic adaptation of effectors was strongly linked to fungal pathogens -host interactions, for both plants and animals, suggesting gene-for-gene relationships is also prevalent in fungus–animal interactions (Shang et al., 2016). In the example of the nematode-trapping fungus *Duddingtonia flagrans*, 117 effectors were predicted, of which PefB was induced during interaction with the host and was imported into host nuclei *in vitro* (Youssar et al., 2019).

A symbiotic lifestyle requires an inducible ability to control plant defenses, a function attributed to mycorrhiza-induced small secreted proteins (MiSSPs) (Plett et al., 2011; Pellegrin et al., 2015; Martin et al., 2016; de Freitas Pereira et al., 2018; Martino et al., 2018). The best-studied example is MiSSP7 in *Laccaria bicolor*, which is upregulated in ectomycorrhizal root tips (Martin et al., 2008). It is secreted into the extracellular environment after sensing of diffusible plant signals and immediately translocated into the root. *In planta*, MiSSP7 manipulates the co-receptor of jasmonate and related signaling pathways of the host, to initiate fungal colonization (Plett et al., 2011, 2014; Martin et al., 2016; Peter et al., 2016). MiSSP8 is one of the highly induced MiSSPs in *L. bicolor* during the early stages of symbiosis. MiSSP8 has a fungal-specific repetitive motif, present not only in ectomycorrhizal but also in saprotrophic SSPs, and might also be relevant for fruiting body formation (Pellegrin et al., 2019a).

The *Rhizophagus irregularis* SP7 protein, manipulates the ethylene-signaling pathway and is localized to the host nucleus where it subsequently reshuffles plant defense pathways (Kloppholz et al., 2011). Gene expression analyses of *Cenococcum geophilum* interacting with different hosts were used to identify MiSSPs and showed that six of them are targeted to distinct subcellular compartments *in planta* (de Freitas Pereira et al., 2018). The fact that SSPs have been found in the genomes of plants that host mycorrhizal fungi (Yang et al., 2011) supports the occurrence of a cross-talk process between fungal MiSSPs and the plant SSPs to establish mutualistic symbiosis. In *Populus trichocarpa*, effector-like SSPs are upregulated during symbiotic interactions. They have been shown to enter *L. bicolor* hyphae, localize to the nucleus and manipulate hyphal growth and morphology (Plett et al., 2017).

Another example of SSPs are hydrophobins are present across the fungal kingdom and have an ability to self-assemble into films at hydrophilic-hydrophobic interfaces (Wessels, 1996; Bayry et al., 2012). They have been found to be regulated during development of the ectomycorrhizal basidiomycete *Tricholoma vaccinum* and differentially expressed during the early steps of mycorrhization, in a manner that is dependent on host preference (Sammer et al., 2016). Hydrophobins and other effector-like proteins are also delivered during *Trichoderma* association with plants (Ramírez-Valdespino et al., 2019).

Interestingly in the *Epichloë* spp. which are capable of beneficial associations, the majority of SSPs were upregulated in mutants with antagonistic associations, while some were significantly downregulated, suggesting a role in controlling the mutualistic/pathogenic states (Eaton et al., 2015). Candidate effectors in *Epichloë festucae* were analyzed in their interaction with *Lolium perenne*, but their function in fungal-host interactions was masked by redundancy with other effectors (Hassing et al., 2019).

## SSPs in Saprotrophic Fungi

SSPs comprise about 40% of the secretomes of saprophytes, but only a few of them have been functionally characterized (Pellegrin et al., 2015). In saprotrophic fungi, SSPs can be involved in degradative capabilities like in the case of *Trichoderma reesei* swollenin, which depolymerizes cellulose (Saloheimo et al., 2002). SSPs could also recruit enzymes at the surface of the substrate or interact with enzymes to increase their activity. For example, HsbA in *Aspergillus oryzae* has been demonstrated to recruit lytic enzymes to the surface of hydrophobic solid materials (Ohtaki et al., 2006). In an analysis comparing secretion patterns of SSPs in 8 *Aspergillus* spp., HsbA and stress-related superoxide were found in the context of plant biomass degradation and were secreted in an *Aspergillus* spp-specific manner. Other SSPs were suggested to be a part of the fungal stress response to the toxicity of several aromatic compounds or reactive oxygen species released during biomass degradation (Valette et al., 2017). In the ligninolytic fungus *Phanerochaete chrysosporium* over 40 SSP-coding genes are induced when grown in the presence of oak extracts, and are suggested to play a role in cell protection/signaling in response to toxic compounds (Thuillier et al., 2014; Fernández-González et al., 2018).

The EffectorP 2.0 program has predicted 12% of the proteins secreted by saprophytes to be effector-like (Sperschneider et al., 2018). Genomic comparisons between pathogenic and saprophytic species were used to reveal the differences between fungi exhibiting the two lifestyles, unveiling an intriguing role of the effector repertoires. The genomic effector range of activity in the saprophytic fungus *Verticillium tricorpus* resembles that of its plant pathogenic relative *V. dahliae* (Seidl et al., 2015). Genome-wide analysis of the transition to pathogenic lifestyles in *Magnaporthales* showed that pathogens had more clade-specific SSPs than the wood-inhabiting non-pathogenic clade. Some of the effectors were specific to the pathogenic clade and are likely to play key roles in the interaction with the host, but some were conserved with those of the saprophytic members (Zhang et al., 2018). In *Fusarium* spp., most putative effectors are conserved among pathogens and saprophytes, but 38 candidate effectors were found only in the pathogenic *Fusarium* spp. (King et al., 2018). Another example is the large arsenal of effectors in *Pseudozyma*, saprotrophic yeasts, which has been suggested to have originated from an unknown plant pathogenic stage required for sexual recombination (Sharma et al., 2019). Thus, while some effectors are specific to pathogenic or mutualistic species and are probably required for interaction with the host, a substantial number of “effector-like” proteins are also present in their saprotrophic relatives and whose functions have yet to be elucidated.

In mushroom-forming fungi, fruiting body secretomes contain a rich suite of genes encoding SSPs (Krizsán et al., 2019), some of which are developmentally regulated (Almási et al., 2019). An impressive portion of the SSPs (20–61%) is developmentally regulated, with ∼20% being conserved across all tested species, and are comprised of fungal cell wall-related families, such as hydrophobins, cerato-platanins, cupredoxins, lectins, Kre9/Knh1, GH12, and LysM domain proteins (Krizsán et al., 2019). Cerato-platanin proteins are believed to be important for plant-fungal interactions but are also present in saprophytes such as *Trichoderma* (Kubicek et al., 2019). Over 40 developmentally-regulated SSPs have yet to be functionally annotated and are currently regarded as species-specific (Krizsán et al., 2019). In *Phlebia radiata*, an efficient plant cell wall decomposer, 430 genes coding for SSPs were found, 83 of which were also identified by EffectorP -2.0. The function of most of these effector-like SSPs is still unknown (Mäkinen et al., 2019).

SSPs may also be involved in fungal-bacterial interactions. SSP gene expression was reported to be induced during interactions between *Podospora anserina* and *Serratia* species (Lamacchia et al., 2016) and in *Coprinopsis cinerea* in response to the presence of bacteria (Kombrink et al., 2019).

## Ssp1 in *P. ostreatus* – A Case Study

*P. ostreatus* is a white-rot edible mushroom, capable of utilizing a variety of organic substrates following their decomposition. The genome of the *P. ostreatus* monokaryotic PC9 strain harbors 534 SSPs, of which 162 were predicted as effectors by the effectorP 2.0 program (Sperschneider et al., 2018). About 30% were species-specific SSPs, similar to the number reported in other fungi (Kim et al., 2016). About 27% of the predicted effector – like proteins in PC9 lack any known annotation and are still designated as hypothetical, promising a potential to yield yet undiscovered functions. Interestingly, some families such as ricin-B-lectin and hydrophobins are conserved in *Pleurotus* like in most fungi included in the analysis. Our analysis is biased toward predicted effectors found in the proteome of Pleurotus, a saprophytic WRF, thus excluding pathogens or ectomycorrhizal specific effectors, hence no apparent clades according to life-style occurred (Supplementary Figure S1). On the other hand the four representative of the Ascomycota, comprise a different clade.

An example of effector-like SSPs with hypothetical annotation is the poSSP family of the PC9 strain. It includes six members, encoding proteins of (∼18 kDa that harbor a signal peptide (Feldman et al., 2017). They were first identified by their upregulation (∼4,500-fold) following exposure to 5-hydroxymethylfurfural (HMF). Similar upregulation occurred after exposure to several other aryl-alcohols as well as during idiophase (after the onset of secondary metabolism) (Feldman et al., 2017). Aryl alcohols function in harmony with ligninolytic enzymes by providing H2O2 to the peroxidases and are even synthesized *de novo* in some WRF (Hernández-Ortega et al., 2012). Ssp1 was also the most upregulated gene, up to 700-fold, during interspecific interactions between *P. ostreatus* with *Dichomitus squalen* or *Trametes versicolor* (Zhong et al., 2017).

Homologs of poSSPs were found in 22 fungal species. Among them, 16 members of the order Agaricales and 3 of Auriculariales. Most of these are basidiomycota and only 3 representatives of the ascomycota were found. A majority of these fungi harbor multiple, highly similar, copies of Ssp1-coding genes (Table 1). This may suggest that the genomic origin of these SSPs is from the Agaricales, and the multiple copies were generated through duplication events. Since homologs of Ssp1 are found in genomes of over 20 fungal species representing both saprophytic and mutualistic lifestyles, it is conceivable that they have overlapping and/or conserved functions.

**TABLE 1:** Homologs of Ssp1 in fungi.

**Picture**	**Organism**	**Copies**	**Order**
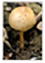	*Agrocybe pediades*	9	Agaricales
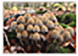	*Coprinellus micaceus*	6	Agaricales
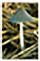	*Coprinopsis cinerea*	8	Agaricales
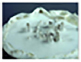	*Coprinopsis cinerea* AmutBmut	4	Agaricales
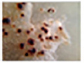	*Coprinopsis sclerotiger*	6	Agaricales
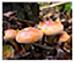	*Hypholoma sublateritium*	3	Agaricales
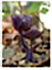	*Laccaria amethystina*	7	Agaricales
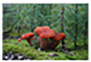	*Laccaria bicolor*	3	Agaricales
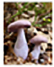	*Lepista nuda*	1	Agaricales
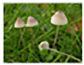	*Mycena filopes* CBHHK001	3	Agaricales
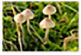	*Mycena floridula* CBHHK072	1	Agaricales
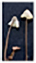	*Mycena rebaudengoi*	2	Agaricales
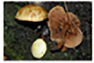	*Pholiota alnicola*	12	Agaricales
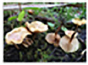	*Pholiota conissans*	5	Agaricales
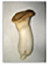	*Pleurotus eryngii*	2	Agaricales
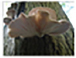	*Pleurotus ostreatus* PC15	5	Agaricales
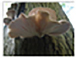	*Pleurotus ostreatus* PC9	6	Agaricales
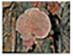	*Auricularia subglabra*	1	Auriculariales
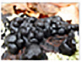	*Exidia glandulosa*	5	Auriculariales
	*Zopfia rhizophila*	1	Incertae sediss
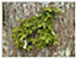	*Lobaria pulmonaria* Scotland	1	Peltigerales
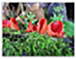	*Sarcoscypha coccinea* ATCC 58028	1	Pezizales

*The identification of homologs was carried out by Blastp (>1.0E−5) on the genomes available at MycoCosm (Grigoriev et al., 2014).*

The wealth of information accumulated concerning the physiology of *P. ostreatus* and its amenability to genetic manipulations (Honda et al., 2000; Salame et al., 2010, 2011, 2012, 2014) have been instrumental in studying the nature of poSSPs. Reverse genetics was used to elucidate the function of Ssp1, by using RNAi to knockdown expression of the entire family (KD*ssp1*) or by overexpression of the dominant Ssp1 member (OE*ssp*1) (Feldman et al., 2017). Analysis of the phenotypes revealed an interesting role of Ssp1 in the regulation of development. The mutations in Ssp1 conferred a time shift in their secretion and expression patterns; OE*ssp1* entered the idiophase earlier, accompanied by the formation of yellow pigment and faster aging. In contrast, the KD*ssp1* had a longer tropohase, the strain did not generate pigmentation and no lysis of the colony occurred. Taken together, Ssp1 was shown to be involved in the transition from tropohase to idiophase, aging, and metabolism (Feldman et al., 2019), along with regulating ligninolytic enzyme production (Feldman et al., 2017). Overexpression of Ssp1 also negatively affected fruiting body initiation (Feldman et al., 2019), which is in line with Krizsán et al. (2019), who suggested that SSPs function as fruiting body effectors. It seems that different poSSPs are regulated and produced under diverse conditions. Ssp1-3 were produced in rich peptone-glucose media and following exposure to HMF (Feldman et al., 2017). However, Ssp6 was produced in cellulose-based media (Yoav et al., 2018).

## Discussion

SSPs are a significant, yet still a functionally-elusive, part of the fungal secretome, especially in saprophytes. Nonetheless, some of them are the likely ancestors of the better understood effectors in pathogenic and mutualistic fungi (Seidl et al., 2015; King et al., 2018; Zhang et al., 2018). Part of the enigma concerning the role of SSPs in saprophytes is based on the alleged contradiction that effector-like SSPs are a considerable component of their secretome, yet, these organisms obtain nutrients directly from dead organic matter without forming interactions with a living host. This raises the question – whom do these effector-like proteins affect? One tempting conclusion is that SSPs participate in the ability of these fungi to grow in a specific ecological niche, yet in a completely unrelated fashion to that elucidated for pathogens.

**FIGURE 1:**
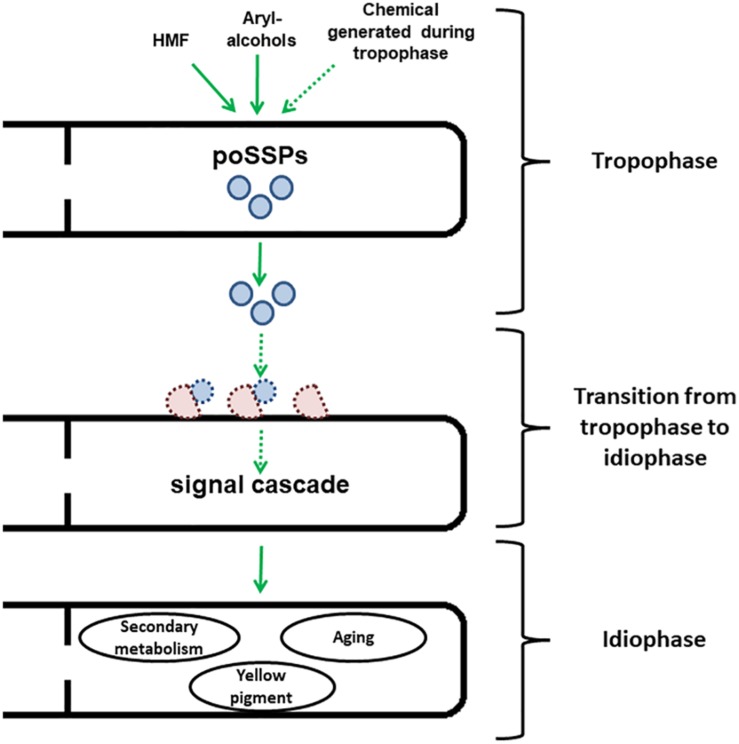
Scheme representing the speculated model for developmental regulation by Ssp1 in *P. ostreatus*. Ssp1 is transcriptionally upregulated after the fungus is either exposed to chemical cues such as HMF or aryl-alcohols or reaching the end of the trophophase. After the cleavage of the signal peptide, they are secreted to the medium. Then, they interact with a yet to be identified transporter to initiate a signal cascade, which results in the onset of idiophase accompanied by secondary metabolism, generation of yellow pigment and aging. Dashed arrows and shapes represent pathways that are yet to be found.

In spite of widespread cytoplasmic continuity present in many filamentous fungi, both environmental changes as well as inherent developmental factors can confer phenotypic heterogeneity along different sections of an integral colony (Tegelaar and Wösten, 2017; Op De Beeck et al., 2020; Tegelaar et al., 2020). Is it possible that SSPs are “self-effectors”, which are involved in the regulation of the mentioned heterogeneity? SSPs have been previously suggested to target and involve communication with microbial co-inhabitants, and not directed only toward “true” hosts (Rovenich et al., 2014). One form of microbial communication is quorum sensing (QS) (Miller and Bassler, 2001). In fungi, QS has been described to regulate processes such as sporulation, secondary metabolite production, morphological transition and enzyme secretion (Barriuso et al., 2018). The effects of Ssp1 in *P. ostreatus* highly resemble the mentioned characteristics of QS (Feldman et al., 2017, 2019). The majority of the known fungal QS molecules are chemical compounds, such as farnesol and alcohol tyrosol, like in *Candida albicans* (Albuquerque and Casadevall, 2012; Franceschetti et al., 2017; Barriuso et al., 2018). However, in the case of *Cryptococcus neoformans*, a human basidiomycete pathogen, QS is regulated via secreted peptides (Homer et al., 2016; Franceschetti et al., 2017). As of yet, only limited supporting evidence is available concerning the roles of SSPs in communication between cells of the same organism, and between hyphae within a colony (Vincent et al., 2012). While still scarce, these data may indicate that Ssp1 indeed could have the potential to function in cell-to-cell communication, and act as a regulator after initiation of signal transduction in a dose-dependent manner (for a proposed model, see Figure 1).

Another intriguing possibility arises from recent evidence on the ability of some saprophytes to exhibit facultative biotrophic attributes. In fact, 34 out of 201 species of wood-decay basidiomycetes were shown to be able to colonize the roots of at least one tree species, supporting the feasibility for facultative biotrophic relationships in some free-living saprotrophs (Smith et al., 2017). Research on the genus *Amanita* showed that the origin of the genetic toolkit required for symbiosis is found in a saprotrophic species (Hess et al., 2018). Could some of these features be attributed to the unknown and partially conserved effectors in the genome of saprophytes? Or, is there a wider overlapping “gray” area between the opposing fungal lifestyles and perhaps a similar, pathogenesis-related function of proteins occurs in saprophytes?

Much of the current research focuses on the genomics and bioinformatics-based analyses of SSPs, which provides much valued phylogenetic and evolutionary perspectives. Various omics approaches may well guide us to the discovery of interesting SSPs, whose functions can now be deciphered with the increase in classical and molecular tools available for fungal research. This is not limited to *P. ostreatus* (as detailed above), but can be carried out in a growing array of SSP-producing fungi, such an *Armillaria ostoyae, Schizophyllum commune*, and more (Krizsán et al., 2019).

A significant challenge concerning the functional analysis of SSPs lays in the possibility that the high diversity of effector protein sequences mask the potentially large scale of their functional redundancy in fungal genomes. It is possible that genes lacking homology at the primary sequence level may still have similar 3D structures and, hence, similar functions (Lo Presti et al., 2015; Plett and Martin, 2015; Selin et al., 2016; Franceschetti et al., 2017; Lo Presti and Kahmann, 2017; Plissonneau et al., 2017). This is also one of the challenges in deciphering the link between SSP structures and functions in ectomycorrhizal interactions (Pellegrin et al., 2019b). Conversely, some SSPs within a given species are extremely similar (e.g., *P. ostreatus*), imposing technical difficulties when it comes to their genetic manipulation. In order to uncover and validate their role(s) and function(s), the research must be accompanied by a “wet bench” experimental approach. Perhaps some of these can be achieved by using RNAi and/or CRISPR-Cas9 technologies (Salame et al., 2011; Shi et al., 2017), or learning their function through labeling and localization, interactions with proteins such as acceptors, and external addition of purified native or heterologously-expressed SSPs, for activity and structural studies.

Overall, effector-like SSPs in saprophytes are one of the less-studied parts of their secretome, but the accumulating evidence points to their importance. As the comprehensive understanding of SSPs is still elusive, it is of interest to draw from what is known and proven, but at the same time suggest additional hypotheses and ideas to inspire the community to expand further the experimental-based analysis of SSPs. Exploring and revealing their roles, will not just improve the current understanding of development and communication in saprophytic fungi, but will also help to elucidate the origin, regulation, and mechanisms of fungal-host, fungal-fungal, and fungal-bacterial (and other microbial) interactions.

## Author Contributions

DF, OY, and YH wrote this mini-review together.

## Conflict of Interest

The authors declare that the research was conducted in the absence of any commercial or financial relationships that could be construed as a potential conflict of interest.

## Supplementary Material

FIGURE S1 Dendrogram of effector-like SSPs in fungi across different lifestyles on the basis of the *P. ostreatus* secretome. The analysis is based on predicted proteins from *P. ostreatus* that are smaller than 300 amino acids, have a signal peptide, and predicted as effectors by the effectorP 2.0 program (Sperschneider et al., 2018). The dendrogram presents the homology percentage of proteins from *P. ostreatus* to proteins from other fungi. The genomic information was obtained from MycoCosm (Grigoriev et al., 2014). The analysis was preformed and visualized with EXPANDER (Hait et al., 2019). Annotation were based on MycoCosm or curated manually. The poSSPs were highlighted. The fungal lifestyle were color labeled pathogenic fungi (pink), symbionts (green), and saprophytes (blue).

## Abbreviations

CAZyme: carbohydrate-Active enZymes
PFAM: protein families
RNAi: RNA interference
SSP: small secreted proteins
WRF: white-rot fungi.
