# Supplementary figures and images for: Seeking the Roles for Fungal Small-Secreted Proteins in Affecting Saprophytic Lifestyles
Source: Front Microbiol. 2020 Mar 24;11:455. doi: 10.3389/fmicb.2020.00455 (PMC7105643; doi:10.3389/fmicb.2020.00455)

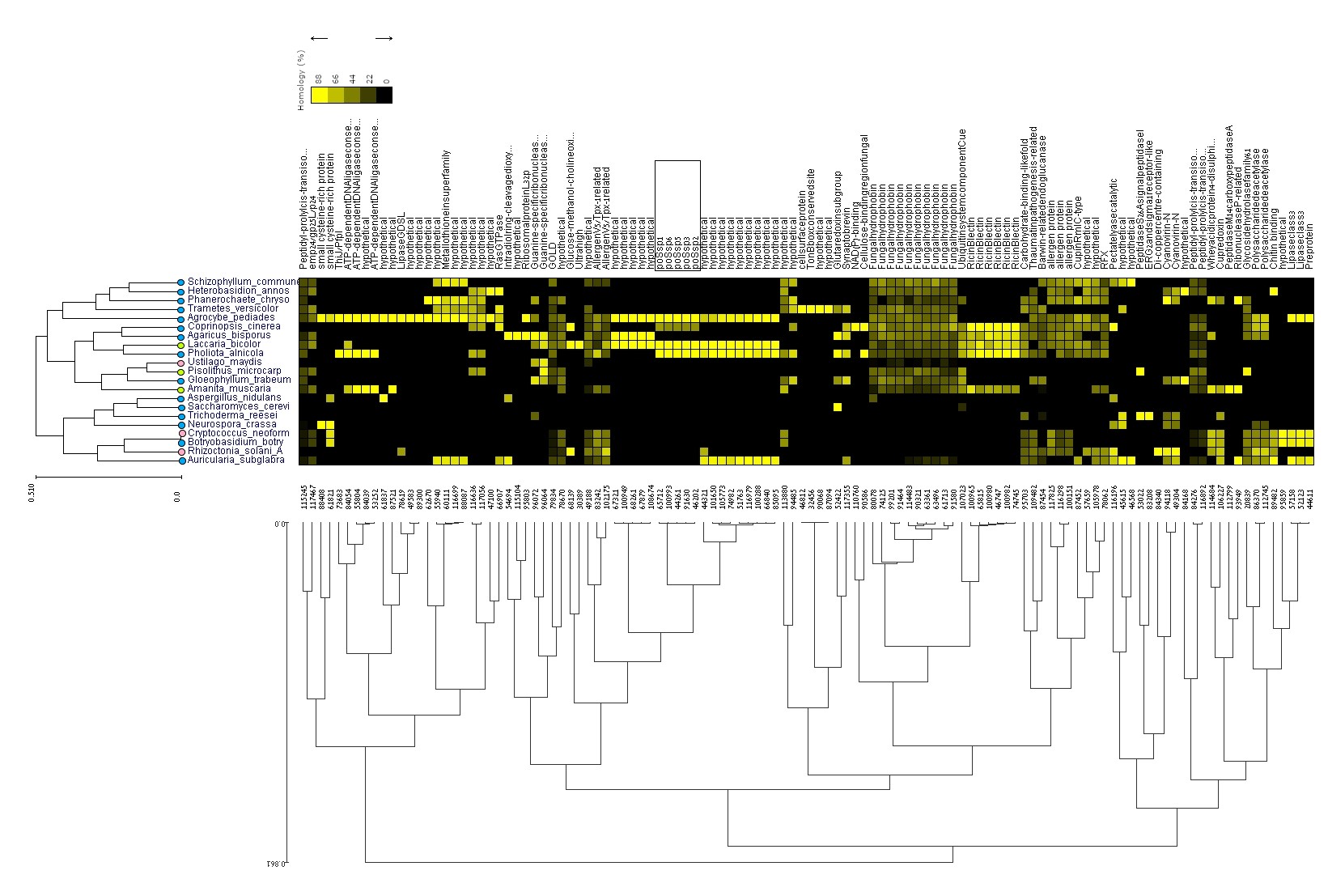

Supplement: FIGURE S1 — Dendrogram of effector-like SSPs in fungi across different lifestyles on the basis of the P. ostreatus secretome. The analysis is based on predicted proteins from P. ostreatus that are smaller than 300 amino acids, have a signal peptide, and predicted as effectors by the effectorP 2.0 program (Sperschneider et al., 2018). The dendrogram presents the homology percentage of proteins from P. ostreatus to proteins from other fungi. The genomic information was obtained from MycoCosm (Grigoriev et al., 2014). The analysis was preformed and visualized with EXPANDER (Hait et al., 2019). Annotation were based on MycoCosm or curated manually. The poSSPs were highlighted. The fungal lifestyle were color labeled pathogenic fungi (pink), symbionts (green), and saprophytes (blue). [file Image_1.JPEG]
